# *In Silico* Approaches Applied to the Study of Peptide Analogs of Ile-Pro-Ile in Relation to Their Dipeptidyl Peptidase IV Inhibitory Properties

**DOI:** 10.3389/fendo.2018.00329

**Published:** 2018-06-14

**Authors:** Alice B. Nongonierma, Luca Dellafiora, Sara Paolella, Gianni Galaverna, Pietro Cozzini, Richard J. FitzGerald

**Affiliations:** ^1^Department of Biological Sciences and Food for Health Ireland (FHI), University of Limerick, Limerick, Ireland; ^2^Food and Drug Department, University of Parma, Parma, Italy

**Keywords:** dipeptidyl peptidase IV inhibition, Ile-Pro-Ile (diprotin A), bioactive peptides, peptide analogs, design of experiments, quantitative structure activity relationship, molecular docking

## Abstract

Inhibition of dipeptidyl peptidase IV (DPP-IV) may be exploited to maintain the incretin effect during the postprandial phase. As a result, glycemic regulation and energy homeostasis may be improved. Food protein-derived peptides have been identified as natural agents capable of inhibiting DPP-IV. Ile-Pro-Ile is the most potent DPP-IV inhibitory peptide identified to date. A minimum analog peptide set approach was used to study peptide analogs of Ile-Pro-Ile. The DPP-IV half maximal inhibitory concentration (IC_50_) values of the 25 peptides evaluated ranged from 3.9 ± 1.0 µM (Ile-Pro-Ile) to 247.0 ± 32.7 µM (Phe-Pro-Phe). The presence of Pro at position 2 of tripeptides was required to achieve high DPP-IV inhibition. Most peptides behaved as competitive inhibitors of DPP-IV with the exception of peptides with a N-terminal Trp, which were mixed-type inhibitors. While possessing the structure of preferred DPP-IV substrates, most peptides studied were particularly stable during 30 min incubation with DPP-IV. Molecular docking revealed that Ile-Pro-Ile and its peptide analogs interacted in a very similar manner with the active site of DPP-IV. In addition, no correlation was found between the Hydropathic INTeraction score and the DPP-IV IC_50_ values of the peptides studied. This outcome suggests that free energy may not be directly responsible for enzyme inhibition by the peptides. Finally, novel DPP-IV inhibitory peptides were identified using the strategy employed herein. These results may be relevant for the development of food protein-derived peptides with serum glucose lowering and food intake regulatory properties in humans.

## Introduction

The inhibition of dipeptidyl peptidase IV (DPP-IV) is one of the numerous therapeutic strategies used for the management of type 2 diabetes. DPP-IV can cleave incretins such as glucagon-like peptide-1 (GLP-1) and glucose inhibitory polypeptide, resulting in a loss of their insulinotropic activity during the postprandial phase. The inhibition of DPP-IV has been shown to result in a better regulation of glycemia in type 2 diabetic subjects (1, 2). For this reason, several DPP-IV inhibitory drugs, known as gliptins, have been developed as antidiabetic agents (1). In addition, GLP-1 has been shown to modulate energy homeostasis by slowing down gastric emptying, inducing satiety, and reducing food intake (3).

Food proteins also contain peptide sequences, which are able to inhibit DPP-IV *in vitro*, in cell cultures and in small animals [for reviews, see Ref. (4, 5)]. Therefore, food protein-derived peptides may have an effect on the regulation of glucose metabolism *in vivo*. Food protein hydrolyzates have often been characterized for their DPP-IV inhibitory potency by quantifying their half maximal inhibitory concentration (IC_50_). Food protein-derived hydrolyzates are generally ~10^4^ times less potent than gliptins (6). However, food and drug interventions may be complimentary in the management of type 2 diabetes (7). In fact, combinations of gliptins and hydrolyzates can have additive effects in terms of their ability to inhibit DPP-IV (6).

To date, the most potent DPP-IV inhibitory peptides reported in the literature are Ile-Pro-Ile and Val-Pro-Leu, also known as diprotin A and B, respectively. Their DPP-IV IC_50_ value is 3.5 and 15.8 µM, respectively (8). Both peptides were originally isolated from *Bacillus cereus* cultures (8). However, Ile-Pro-Ile and Val-Pro-Leu are also present in the primary sequence of several food proteins (9). The details of particularly potent DPP-IV inhibitory peptides displaying IC_50_ values < 100 µM have recently been reviewed (5). Ile-Pro-Ile and Val-Pro-Leu were, respectively, shown to be >12 and 2 times more potent than other food protein-derived peptides. In addition, only nine other tripeptides having DPP-IV IC_50_ values < 100 µM have been identified to date (Table S1 in Supplementary Material). Interestingly, all these tripeptides possess a Pro at position 2. This suggests that peptides having the structure Xaa_1_-Pro-Xaa_3_ (with Xaa an amino acid residue), may be of interest in studying those properties, which dictate the potency of DPP-IV inhibitory peptides. Furthermore, Pro-containing peptides have been reported to be particularly resistant to gastrointestinal digestion and potentially bioavailable as demonstrated by their detection in the gastrointestinal tract and in the circulation of humans [for reviews, see Ref. (10–12)].

Analogs of lead drug compounds are often used in drug discovery to design molecules with higher activity, improved bioavailability, or reduced side effects (13). DPP-IV inhibitory peptide analogs possessing the structure Trp-Arg-Xaa were studied using a peptide library of the 20 possible peptides (14). The most potent peptide discovered using this approach was Trp-Arg-Glu having a DPP-IV IC_50_ value of 350 µM. However, the utilization of a peptide library strategy to study Xaa_1_-Pro-Xaa_3_ would involve the analysis of 400 peptides (20 × 20 amino acids for Xaa_1_ and Xaa_3_). Therefore, in order to reduce the number of peptides for analysis, the concept of minimum analog peptide set (MAPS) has been introduced (15, 16). MAPS has been described during the study of peptide analogs employed for the development of meaningful quantitative structure activity relationship (QSAR) models for angiotensin converting enzyme (ACE) inhibition. MAPS correspond to specific peptide sets containing a minimum number of sequences, which are chosen by factorial or fractional factorial design to incorporate physicochemical properties relevant for a target bioactivity. To date, MAPS has been employed in the prediction of oxytocin (oxytocic and pressor activity), pepstatin (inhibition of porcine pepsin), and bradykinin (bradykinin potentiating) analogs (16). MAPS approaches do not appear to have been widely employed for the study of food protein-derived bioactive peptides.

The aim of this study was, therefore, to apply a MAPS-based approach to design peptide analogs of Ile-Pro-Ile. This was achieved by developing a QSAR model for DPP-IV inhibitory peptides to better understand the physicochemical characteristics, which are relevant to DPP-IV inhibition. In a second stage, a factorial design was employed to design the peptide analogs. The peptides were then assessed for their *in vitro* DPP-IV inhibitory properties and their mode of inhibition. Finally, molecular docking studies were conducted to better understand how those peptides may interact with the active site of DPP-IV.

## Materials and Methods

### Reagents

High-performance liquid chromatography (HPLC) grade water was from VWR (Dublin, Ireland). Trifluoroacetic acid, formic acid (FA), tris(hydroxymethyl)aminomethane (Tris), Gly-Pro-pNA, diprotin A (Ile-Pro-Ile), porcine DPP-IV (≥10 U mg^−1^ protein), mass spectrometry (MS) grade water, and acetonitrile were purchased from Sigma Aldrich (Dublin, Ireland). Synthetic peptides (Ala-Ile-Pro, Ala-Pro-Ala, Ala-Pro-Phe, Ala-Pro-Arg, Ala-Arg-Pro, Ile-Phe-Lys, Ile-Ile-Phe, Ile-Pro-Ala, Ile-Arg-Phe, Ile-Arg-Lys, Lys-Pro-Ala, Lys-Arg-Ile, Arg-Ile-Phe, Arg-Ile-Arg, Arg-Lys-Arg, Phe-Pro-Phe, Phe-Pro-Ile, Phe-Pro-Trp, Ile-Ala-Ile, Ile-Pro-Phe, Ile-Pro-Trp, Trp-Pro-Phe, Trp-Pro-Ile, Trp-Pro-Trp) with a purity > 95% (w/w) were obtained from Genscript (Piscataway, NJ, USA).

### QSAR Modeling

The QSAR analysis linking the DPP-IV IC_50_ value of peptides to their physicochemical properties was conducted using a partial least squares regression (PLSR) model (Eq. 1) as previously described (17). The peptides used (Table 1) were all competitive inhibitors of DPP-IV, and their IC_50_ values were obtained using the same DPP-IV inhibition assay (18). These conditions were selected given that it was previously demonstrated that this was required in order to obtain a significant PLSR model (17). The structural *v*-scale [*v*_1_ (van der Waals volume), *v*_2_ (net charge index), and *v*_3_ (hydrophobic parameter of side chains)] from Lin et al. (19) was employed for the amino acid descriptors. Peptide descriptors for the two N- and C-terminal amino acids of the sequences were generated to take account of peptides with various lengths (2–9 amino acids, Table 1) as described earlier (20). The PLSR model employed is described in Eq. 1

(1) $$Y=c+\sum_{1}^{i}\sum_{1}^{j}a_{i,j}N_{i,j}{\;}_{\;}+\sum_{1}^{i}\sum_{1}^{j}b_{i,j}C_{i,j}+\varepsilon$$

with *Y*: the Log_10_(DPP-IV IC_50_); *i*: the N- or C- terminal position of the amino acid (1 or 2); *j*: the amino acid descriptor number (varying between 1 and 3); *a* and *b*: the coefficients of the model; *c*, and ε the constant and residual of the model, respectively.

**Table 1:** Dipeptidyl peptidase IV (DPP-IV) half maximal inhibitory concentration (IC_50_) of competitive inhibitory peptides used in the quantitative structure activity relationship model.

Peptide sequence^a^	Length	DPP-IV IC_50_ (μM)	Reference
IPI	3	3.5	(21)
IPIQY	5	35.2	(21)
WL	2	43.6	(22)
LPVPQ	5	43.7	(17)
FLQP	4	65.3	(23)
IPM	3	79.4	(17)
LPVP	4	87.0	(24)
MPVQA	5	93.3	(24)
LPYPY	5	108.3	(21)
HL	2	143.2	(18)
IP	2	149.6	(21)
VA	2	168.2	(18)
LLQLEAIR	8	177.8	(24)
YPYY	4	194.4	(21)
LPQNIPPLT	9	205.2	(17)
VPGEIVE	7	224.5	(17)
LPL	3	241.4	(21)
YPY	3	243.7	(21)
LPLPL	5	325.0	(21)
LPLPLL	6	371.5	(17)
FL	2	399.6	(18)
IPSK	4	406.8	(17)
VLGP	4	580.4	(23)
RP	2	657.2	(17)
YP	2	658.1	(21)
LP	2	712.5	(21)
ILELA	5	721.1	(24)
AL	2	882.1	(18)
LW	2	993.4	(22)
QPLPPT	6	1,013.8	(17)
SL	2	2,517.1	(18)
GL	2	2,615.0	(18)
EK	2	3,216.8	(18)

*^a^Peptide sequences abbreviated with the one letter amino acid code. Peptides are ordered by decreasing DPP-IV inhibitory potency*.

Generation of the peptide descriptors and PLSR model was carried out using Matlab (version R2015b, MathWorks, Inc., Natick, MA, USA). Training and test sets comprising 28 and 5 peptides, respectively, were randomly chosen (100 times) by the Matlab algorithm in order to cross validate the PLSR model.

### Experimental Design for the Selection of Peptide Analogs of Ile-Pro-Ile

All the Ile-Pro-Ile analogs used had a fixed length of three amino acids. In addition, the QSAR model used herein, as well as a previous QSAR model (17), highlighted the importance of the amino acid hydrophobicity in the inhibition of DPP-IV. Therefore, a factorial design consisting of 3 factors at 2 levels was employed to design peptide analogs of Ile-Pro-Ile. Each factor represented a position within the peptide sequence (Xaa_1_, Xaa_2_, and Xaa_3_, where Xaa_i_ designates the amino acid located at position i of the peptide) and each level corresponded to a low (−1) or high (+1) hydrophobicity parameter of side chains (*v*_3_) for the amino acid. The 15 conditions of the factorial design corresponding to the hydrophobicity of the selected peptides (Ala-Ile-Pro, Ala-Pro-Ala, Ala-Pro-Phe, Ala-Pro-Arg, Ala-Arg-Pro, Ile-Phe-Lys, Ile-Ile-Phe, Ile-Pro-Ala, Ile-Arg-Phe, Ile-Arg-Lys, Lys-Pro-Ala, Lys-Arg-Ile, Arg-Ile-Phe, Arg-Ile-Arg, and Arg-Lys-Arg), are presented in Table 2.

**Table 2:** Dipeptidyl peptidase IV (DPP-IV) half maximal inhibitory concentration (IC_50_), *z*-centered value of the hydrophobic parameter of side chains [*v*_3_ in the scale developed by Lin et al. (19)] of the amino acids constitutive of the peptides, and mode of inhibition of peptide analog of Ile-Pro-Ile.

Set number	Peptide sequence^a^	*z*-Centered values of*v*_3_ of the amino acids within the tripeptide			Predicted DPP-IV IC_50_ (μM)	Experimental DPP-IV IC_50_ (μM)	Mode of inhibition	Peptide hydrolyzed by DPP-IV

		Xaa_1_	Xaa_2_	Xaa_3_				
1	IPI	0.725	0.059	0.725	238	3.9 ± 1.0	Competitive	NO
	AIP	−0.194	0.725	0.059	103	nd	nd	nd
	APA	−0.194	0.059	−0.194	988	44.3 ± 3.2	Competitive	NO
	APF	−0.194	0.059	0.716	168	65.8 ± 5.5	Competitive	NO
	APR	−0.194	0.059	−1.000	15	119.7 ± 3.5	Competitive	NO
	ARP	−0.194	−1.000	0.059	687	nd	nd	nd
	IFK	0.725	0.716	−0.991	34	nd	nd	nd
	IIF	0.725	0.725	0.716	71	nd	nd	nd
	IPA	0.725	0.059	−0.194	472	28.3 ± 3.4	Competitive	NO
	IRF	0.725	−1.000	0.716	476	nd	nd	nd
	IRK	0.725	−1.000	−0.991	107	nd	nd	nd
	KPA	−0.991	0.059	−0.194	22,690	74.5 ± 7.0	Competitive	NO
	KRI	−0.991	−1.000	0.725	67,699	nd	nd	nd
	RIF	−1.000	0.725	0.716	7,435	nd	nd	nd
	RIR	−1.000	0.725	−1.000	662	nd	nd	nd
	RKR	−1.000	−0.991	−1.000	2,451	nd	nd	nd

2	FPF	0.716	0.059	0.716	177	247.0 ± 32.7	Competitive	YES
	FPI	0.716	0.059	0.725	524	45.2 ± 5.3	Competitive	NO
	FPW	0.716	0.059	1.000	67	54.9 ± 2.9	Competitive	YES
	IAI	0.725	−0.194	0.725	97	nd	nd	nd
	IPF	0.725	0.059	0.716	80	47.3 ± 12.3	Competitive	YES
	IPW	0.725	0.059	1.000	31	175.3 ± 5.5	Competitive	YES
	WPF	1.000	0.059	0.716	214	159.8 ± 12.8	Mixed-type	YES
	WPI	1.000	0.059	0.725	633	133.0 ± 14.7	Mixed-type	NO
	WPW	1.000	0.059	1.000	81	120.1 ± 13.1	Mixed-type	YES

*^a^Peptide sequences abbreviated with the one letter amino acid code. The first set corresponds to Ile-Pro-Ile and its 15 associated peptide analogs. The second set corresponds to Ile-Ala-Ile and the eight additional peptides tested, which possess the Xaa_1_-Pro-Xaa_3_ structure, with Xaa_1_ and Xaa_3_ being Ile, Phe, or Trp*.

*nd, not determined; IC_50_, DPP-IV half maximal inhibitory concentration; NO, peptide not cleaved during incubation with DPP-IV; YES, peptide cleaved following incubation with DPP-IV*.

The experimental values obtained with the 15 peptides were used to develop a predictive multilinear regression (MLR) model linking the % DPP-IV inhibition obtained at 500 µM with the *v*_3_ values of the amino acids contained in the peptides. The MLR is described in Eq. 2

(2) % DPP−IV inhibition=β0+β1x+β2y+β3z+β4x2+β5y2+β6z2+β7xy+β8xz+β9yz+ε

with β_0_ to β_9_: the coefficients of the model; *x, y*, and *z*, the *z*-centered parameters for the *v*_3_ values of Xaa_1_, Xaa_2_, and Xaa_3_, respectively; ε: the residual of the model.

Equation 2 was used to predict the structures of nine tripeptides potentially displaying high DPP-IV inhibition at 500 µM.

### DPP-IV Inhibition Assay and Mode of Inhibition

Peptides were dispersed in HPLC grade water at 50 and 500 µM (final concentration). The DPP-IV inhibition assay was carried out in triplicate as outlined by Nongonierma and FitzGerald (18). Briefly, samples (25 μL) were mixed with Gly-Pro-pNA (final concentration 0.200 mM) and DPP-IV (final concentration 0.0025 U mL^−1^) in a 96-well microplate (Sarstedt, Dublin, Ireland). The microplate was incubated at 37°C for 60 min in a microplate reader (Biotek Synergy HT, Winooski, VT, USA) and absorbance was monitored at 405 nm.

Dose–response curves were determined for peptides displaying a percentage of inhibition > 50% at 500 µM. Peptides were diluted in HPLC water at concentrations ranging from 0.37 to 500 µM (final concentration). The DPP-IV IC_50_ values were determined by plotting the percentage inhibition as a function of the test compound concentration. Each analysis was carried out in triplicate (*n* = 3).

The mode of DPP-IV inhibition was determined using the Lineweaver and Burke double reciprocal representation as previously described (18). The most potent DPP-IV inhibitory peptides (i.e., Ala-Pro-Ala, Ala-Pro-Phe, Ala-Pro-Arg, Ile-Pro-Ala, Lys-Pro-Ala, Phe-Pro-Phe, Phe-Pro-Ile, Phe-Pro-Trp, Ile-Pro-Phe, Ile-Pro-Trp, Trp-Pro-Phe, Trp-Pro-Ile, Trp-Pro-Trp) were assayed at three different final concentrations corresponding to their IC_50_ value divided by 4, 8, and 16. The peptide solution was substituted with 100 mM Tris–HCl buffer pH 8.0 in the negative control. Gly-Pro-pNA was added at concentrations ranging from 0.1 to 0.6 mM (final concentration). The absorbance at 405 nm was recorded for 30 min at 37°C using a microplate reader (Biotek Synergy HT). Each sample was evaluated four times.

### Stability of Peptides to DPP-IV

Stability of the peptides to hydrolysis by DPP-IV was evaluated by liquid chromatography tandem mass spectrometry (LC-MS/MS) using the samples generated during the Lineweaver and Burk analysis containing DPP-IV inhibitory peptides at their IC_50_ values divided by 4 as previously described (24). Following the 30 min reaction, DPP-IV was heat inactivated by immersing samples in a water bath set at 90°C for 20 min. Test samples were subsequently diluted 40 times in mobile phase A (0.1% FA in MS water). Intact peptides were analyzed at the same concentration as the test samples. The peptide composition of the samples was determined as described previously (25). Briefly, an ultra-high performance liquid chromatography (UHPLC) UltiMate 3000 (Dionex, Camberley, Surrey, UK) fitted with a security guard UHPLC C18 PEPTIDE (2.1 mm, Phenomenex, Cheshire, UK) and an Aeris Peptide XB-C18 column (150 mm × 2.1 mm, 1.7 µm, Phenomenex) was used for peptide separation at 25°C. A sample volume of 2 µL was injected with a solvent delay of 4 min. The UHPLC was coupled to a quadrupole time-of-flight mass spectrometer (Q-TOF, Impact HD™, Bruker Daltonics GmbH, Bremen, Germany) using a 50–600 m/z acquisition range. The MS was fitted with an electrospray ionization source used in positive ion mode. Data acquisition was performed with Hystar software (Bruker Daltonics). Data analysis was performed with Compass Data Analysis 4.2 (Bruker Daltonics).

### Molecular Docking of Peptides to the Active Site of DPP-IV

Interactions between the most potent DPP-IV inhibitory peptides and the active site of DPP-IV were investigated using molecular modeling. This analysis was based on docking simulations coupled with rescoring procedures to predict protein–peptide interactions and pharmacophoric analyses to investigate the hydrophobic/hydrophilic properties of the binding site.

The molecular model for porcine DPP-IV was derived from the crystallographic structure deposited in the RCSB Protein Data Bank^1^ having the PDB code 1ORW (26). The protein and peptides analyzed were processed using the Sybyl software, version 8.1^2^ checking the consistency of atom and bond type assignment. The co-crystallized ligands were removed before proceeding with the analysis.

Specifically, the coupling of GOLD, as docking software, and Hydropathic INTeraction (HINT) (27) as rescoring function, was chosen. This was done on the basis of previous studies (i) demonstrating the high reliability of HINT in predicting ligand interactions with several protein targets (28), (ii) estimating the free energy of protein–ligand complex formation (29), and (iii) estimating enzyme inhibitory activity of small molecules and peptides (30, 31). In particular, the HINT score provides an empirical and quantitative evaluation of protein–ligand interaction as a sum of all single atomic contributions. The HINT score correlates with the free energy of binding. Therefore, the higher the HINT score, the more favorable the protein–ligand interaction. The software settings and the docking protocol reported by Dellafiora et al. (31) were used. Briefly, the protein was kept semi-flexible with the polar hydrogens set free to rotate, while ligands were set to be fully flexible. The maximum number of poses generated for each ligand was set at 25, and all the poses underwent a rescoring procedure with the HINT scoring function. Only the pose with the highest HINT score was kept for each ligand as it was considered to be the most favorable.

The pharmacophoric analysis of the ligand binding site was carried out using the Flapsite tool of the FLAP (Fingerprint for Ligand and Protein) software developed by Molecular Discovery Ltd.^3^ (32). The GRID algorithm was used to investigate the corresponding pharmacophoric space (33). The DRY probe was used to describe potential hydrophobic interactions, while the sp^2^ carbonyl oxygen (O) and the neutral flat amino (N1) probes were used to describe the hydrogen bond acceptor and donor capacity of the target, respectively. All images were obtained using the PyMol software version 2.0.^4^

### Statistical Analysis

The significance of the PLSR used in the QSAR modeling (Eq. 1) was assessed using *p* model, *p* lack of fit, *R*^2^ and *R*^2^ cross validation as previously described (17). All statistical analyses were carried out with Matlab.

## Results

### QSAR Model for DPP-IV Inhibitory Peptides

**Figure 1:**
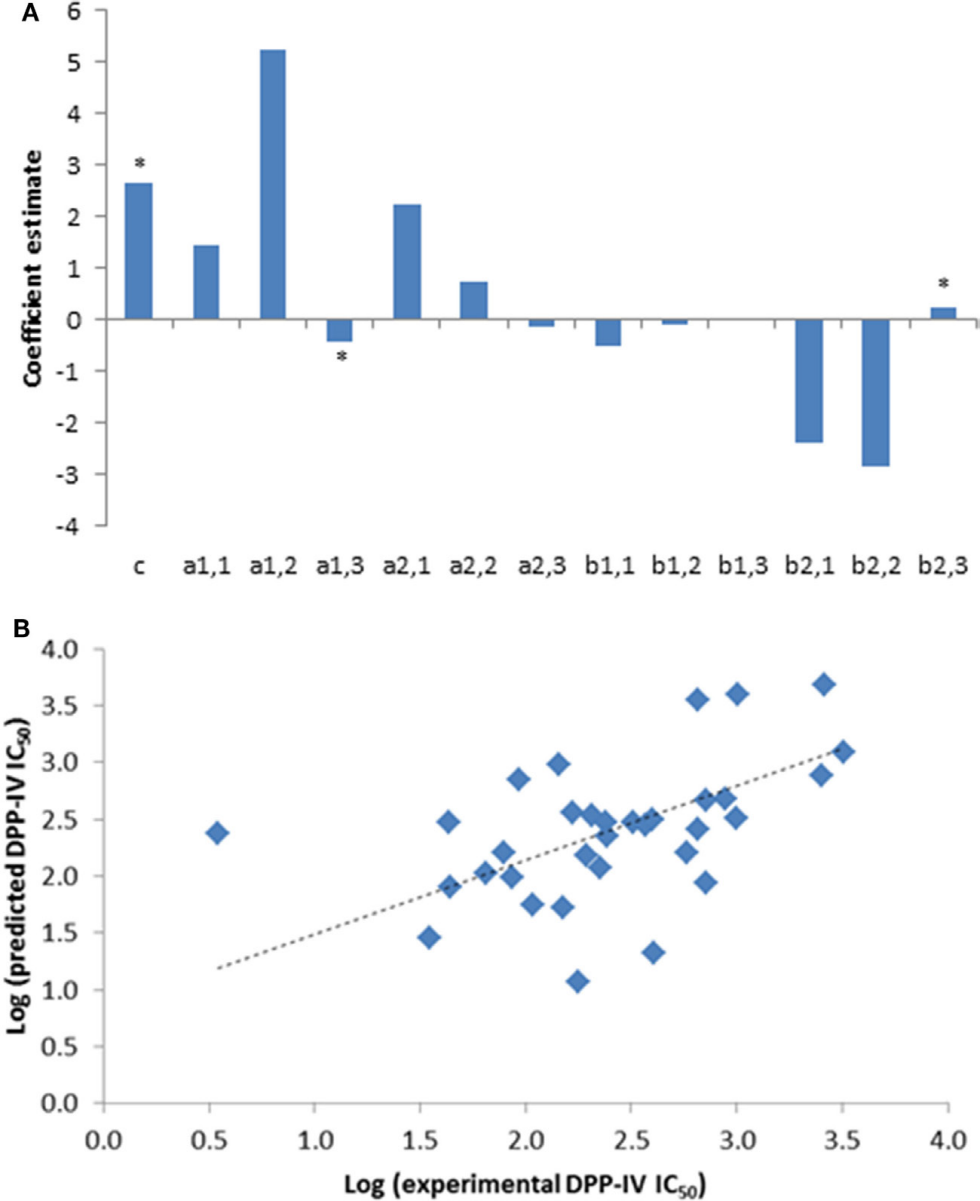
**(A)** Coefficients of the partial least squares regression (PLSR) linking the dipeptidyl peptidase IV (DPP-IV) half maximal inhibitory concentration (IC_50_) of the peptides used to generate the quantitative structure activity relationship (QSAR) model (Eq. 1). *Coefficients with a *p* < 0.05 are significantly different from 0. **(B)** Predicted DPP-IV IC_50_ value of the peptides used to build the QSAR model (Table 1) as a function of the experimental DPP-IV IC_50_ value, the line represents the QSAR model (Eq. 1).

A total of 33 competitive DPP-IV inhibitory peptides were employed to build the PLSR model (Eq. 1; Table 1). The experimentally determined DPP-IV IC_50_ values of these peptides varied between 3.5 and 3,216.8 µM for Ile-Pro-Ile and Glu-Lys, respectively. The PLSR analysis linking the DPP-IV IC_50_ value to the amino acid descriptors of the peptides was statistically significant with a *p* model and *p* lack of fit value of 0.01 and 0.15, respectively (Table 3). The coefficients of the PLSR model, which were significant (*p* < 0.05) were the constant (*c*), *a*_1,3_ and *b*_2,3_ (Figure 1A). This indicated that the hydrophobicity of the amino acids located at the N-terminal and next to the C-terminal positions of peptides were important for the DPP-IV inhibitory potency of peptides. The relationship (*R*^2^ and *R*^2^ cross validation 0.66 and 0.57, respectively, Table 3) between the predicted and experimental DPP-IV IC_50_ value of the peptides used to build the QSAR model (Table 1) is illustrated on Figure 1B.

**Table 3:** Statistical significance of the partial least squares regression linking the dipeptidyl peptidase IV (DPP-IV) half maximal inhibitory concentration (IC_50_) of the peptides used to generate the quantitative structure activity relationship model.

Parameter	value
*n*	33
*R*^2^ model	0.66
*p* model	0.01
Root mean square error	0.45
*F*	3.16
*R*^2^ cross validation	0.57
*p* lack of fit	0.15

### DPP-IV Inhibitory Properties of Peptide Analogs of Ile-Pro-Ile

**Figure 2:**
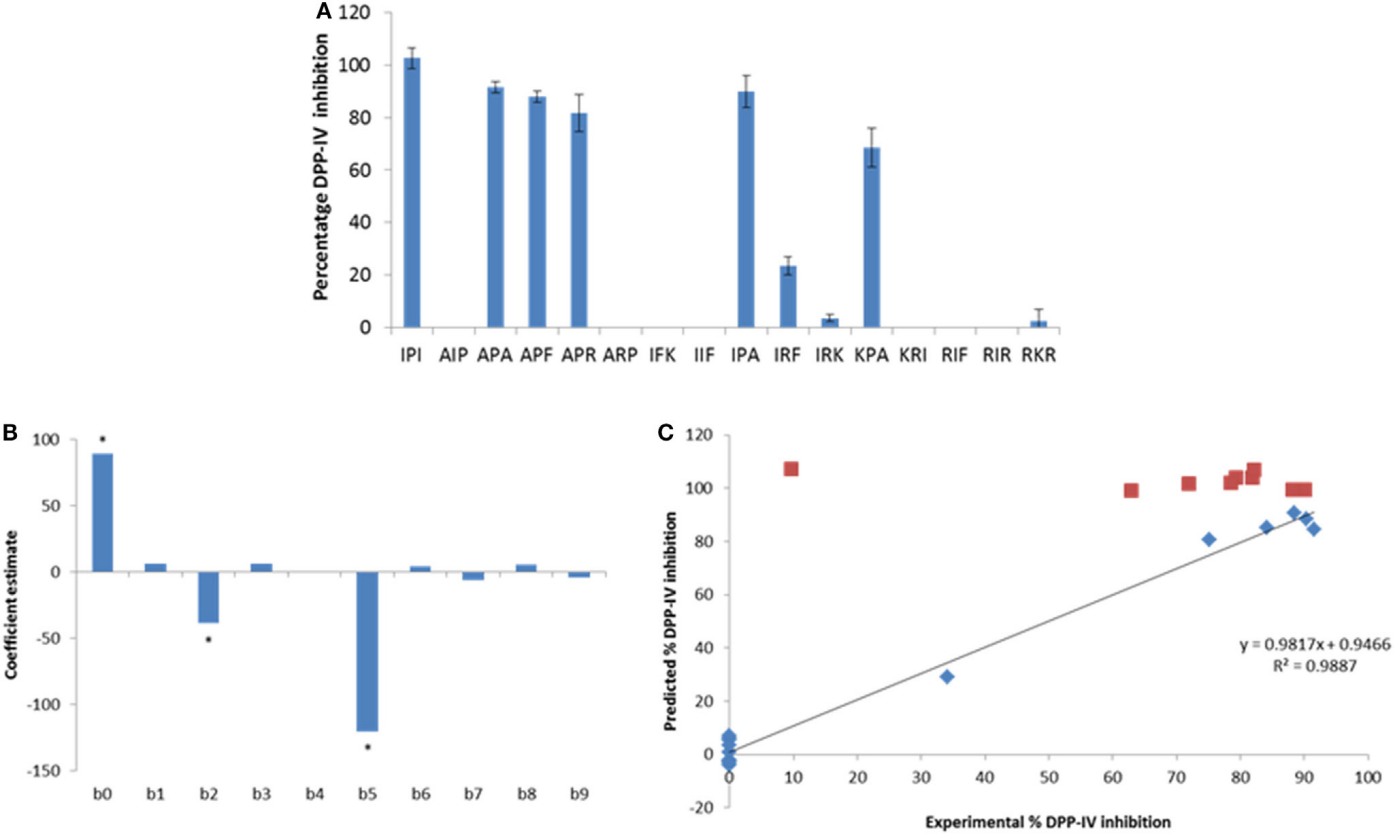
**(A)** Percentage of dipeptidyl peptidase IV (DPP-IV) inhibition observed with the peptides of the minimum analog peptide set evaluated at 500 µM. Values are mean ± SD (*n* = 3). **(B)** Coefficients of the model (β_0_–β_9_; Eq. 2) linking the % DPP-IV inhibition of the peptides at 500 µM to their hydrophobicity (*v*_3_). **(C)** Predicted vs. experimental % DPP-IV inhibition of peptides from set #1 (diamonds) and set #2 (squares) evaluated at 500 µM. The line represents the multilinear regression model determined with data from peptide set #1 (Eq. 2).

Peptide analogs of Ile-Pro-Ile were designed by varying the hydrophobicity of the constitutive amino acids of the tripeptide following a design of experiments (DOE, Table 2). The % DPP-IV inhibition of the 15 tripeptides when evaluated at 500 µM varied depending on the peptide sequence (Figure 2A). Some peptides (Ala-Ile-Pro, Ala-Arg-Pro, Ile-Phe-Lys, Ile-Ile-Phe, Lys-Arg-Ile, Arg-Ile-Phe, and Arg-Ile-Arg) were not able to inhibit DPP-IV at 500 µM. Arg-Lys-Arg, Ile-Arg-Lys, and Ile-Arg-Phe inhibited DPP-IV by less than 25%. The percentage of DPP-IV inhibition observed with the other peptides (Ile-Pro-Ile, Ala-Pro-Ala, Ala-Pro-Phe, Ala-Pro-Arg, Ile-Pro-Ala, and Lys-Pro-Ala) when evaluated at 500 µM was > 65% (Figure 2A).

Equation 2 was used to predict the % DPP-IV inhibition at 500 µM of the 15 tripeptides from set #1. Two coefficients, β_2_ and β_5_, which were associated with the hydrophobicity of the second amino acid of the tripeptides, were significant (*p* < 0.05, Figure 2B). Peptides with an Ala/Thr/His/Gly/Pro at position 2 were predicted to yield high DPP-IV inhibition when evaluated at 500 µM. Pro at position 2 of peptides has been identified as a feature of potent tripeptides (5). Equation 2 predicted that eight tripeptides (Phe-Pro-Phe, Phe-Pro-Ile, Phe-Pro-Trp, Ile-Pro-Phe, Ile-Pro-Trp, Trp-Pro-Phe, Trp-Pro-Ile, and Trp-Pro-Trp), which had the Xaa_1_-Pro-Xaa_3_ structure and similar hydrophobicities as Ile-Pro-Ile would yield high DPP-IV inhibition at 500 µM. Ile-Ala-Ile was also evaluated as Ala has been described as being a preferred amino acid residue at position 2 in DPP-IV peptide substrates (Table 2). The % DPP-IV inhibition at 500 µM of these nine sequences (peptide set #2) is illustrated in Figure 2C. The % inhibition of DPP-IV for these nine sequences was predicted to be >99% using the MLR model (Figure 2C). However, Ile-Ala-Ile could only inhibit 9.6 ± 1.9% of DPP-IV activity when evaluated at 500 µM (Figure 2C). As this peptide was not potent, it was not further evaluated. The % inhibition of DPP-IV by the eight other tripeptides from set #2 varied between 62.9 ± 10.1 and 90.1 ± 1.8% for Phe-Pro-Phe and Phe-Pro-Ile, respectively (Figure 2C).

The IC_50_ values of the most potent peptides, which inhibited DPP-IV by >50% at 500 µM, were determined (Table 2). The QSAR model predicted and the experimentally determined DPP-IV IC_50_ values of the peptides are shown in Table 2. The DPP-IV IC_50_ values of the peptides varied between 3.9 ± 1.0 and 247.0 ± 32.7 µM for Ile-Pro-Ile and Phe-Pro-Phe, respectively (Table 2). The QSAR analysis predicted DPP-IV IC_50_ values for the peptides ranging from 15 to 67,699 µM for Ala-Pro-Arg and Lys-Arg-Ile, respectively (Table 2).

### Mode of DPP-IV Inhibition and Stability of Peptides to DPP-IV

**Figure 3:**
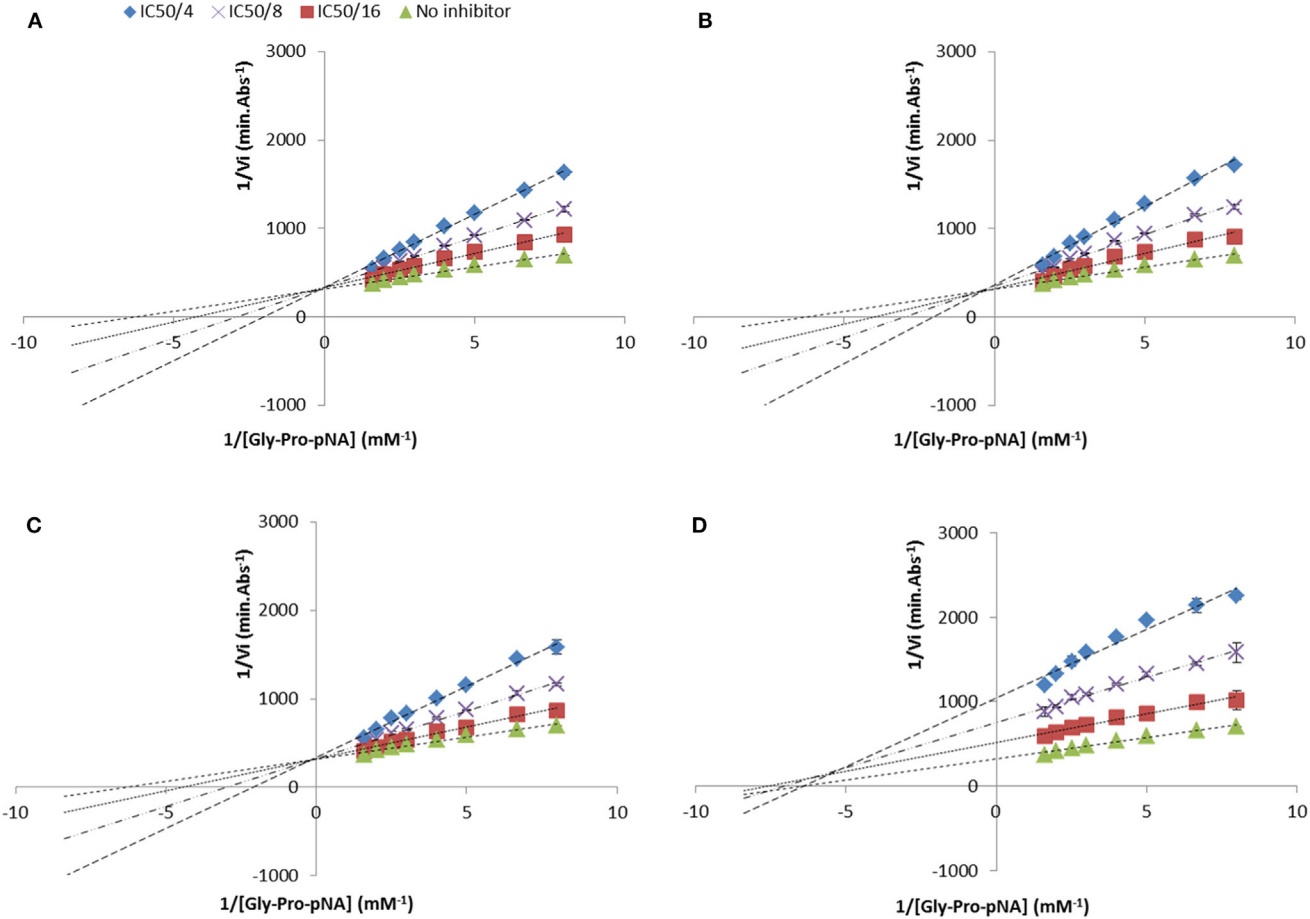
Lineweaver and Burke double reciprocal representation of **(A)** Ile-Pro-Ile (competitive), **(B)** Ala-Pro-Phe (competitive), **(C)** Ile-Pro-Trp (competitive), and **(D)** Trp-Pro-Trp (mixed-type) evaluated at their half maximal inhibitory concentration divided by 4, 8, and 16. Values are the mean reciprocal initial rate ± SD (*n* = 4).

The mode of inhibition of DPP-IV was determined using Lineweaver and Burke double reciprocal plots (Figure 3). Most of the peptides studied in relation to their mode of inhibition (Ile-Pro-Ile, Ala-Pro-Ala, Ala-Pro-Phe, Ala-Pro-Arg, Ile-Pro-Ala, Lys-Pro-Ala, Phe-Pro-Phe, Phe-Pro-Ile, Phe-Pro-Trp, Ile-Pro-Phe, and Ile-Pro-Trp) were competitive inhibitors of DPP-IV. However, three peptides (Trp-Pro-Phe, Trp-Pro-Ile, and Trp-Pro-Trp) were mixed-type inhibitors of DPP-IV (Table 2).

**Figure 4:**
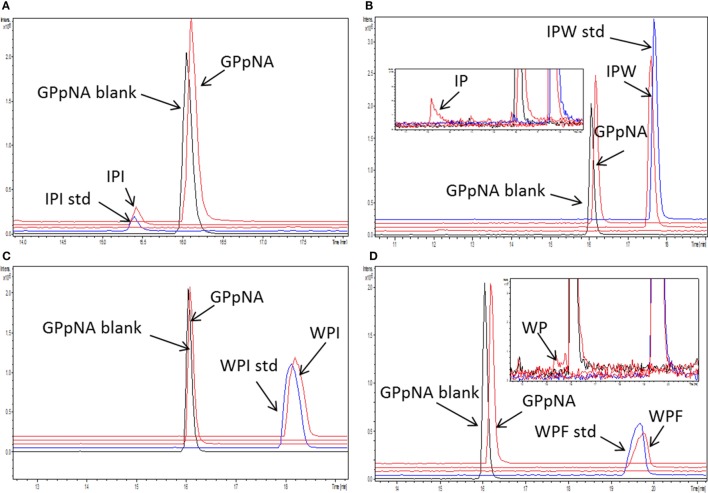
Stability of the peptides [concentration 1/4 of their half maximal inhibitory concentration (IC_50_)] during incubation with dipeptidyl peptidase IV (DPP-IV) and Gly-Pro-pNA for 30 min at 37°C in Tris–HCl buffer pH 8.0. Extracted ion chromatogram of the ultra-high performance liquid chromatography tandem mass spectrometry (MS/MS) of **(A)** Ile-Pro-Ile, **(B)** Ile-Pro-Trp, **(C)** Trp-Pro-Ile, and **(D)** Trp-Pro-Phe. peptide_std: peptide, which was not incubated with DPP-IV; GPpNA_blank: Gly-Pro-pNA incubated with DPP-IV; peptides are abbreviated with the one letter amino acid code.

Peptide stability to DPP-IV was evaluated with the samples generated during the analysis of the mode of inhibition. Several peptides (Ile-Pro-Ile, Ala-Pro-Ala, Ala-Pro-Phe, Ala-Pro-Arg, Ile-Pro-Ala, Lys-Pro-Ala, Phe-Pro-Ile, and Trp-Pro-Ile) were not hydrolyzed by DPP-IV (Table 2; Figures 4A,C). Six peptides (Phe-Pro-Phe, Phe-Pro-Trp, Ile-Pro-Phe, Ile-Pro-Trp, Trp-Pro-Phe, and Trp-Pro-Trp) were partly hydrolyzed by DPP-IV following cleavage at the C-terminal side of the Pro residue (Table 2; Figures 4B,D).

### Molecular Modeling of DPP-IV Binding Interactions With Peptides

The geometry of binding of the most potent peptides within the DPP-IV binding site was investigated using molecular modeling based on pharmacophoric analysis and docking simulations coupled to rescoring procedures. The workflow used has already proved successful in computing the binding architecture of low-molecular mass ligands (34, 35). However, a fit-for-purpose validation was carried out to check the case-specific reliability. To this end, the 3D structures of porcine DPP-IV bound with non-covalent competitive inhibitors, which are available in the RCSB PDB database (36) (see text footnote 1), were taken as validation reference. Specifically, four structures were found (database last accessed on January 25th 2018). The chemical structures of the inhibitors are reported in Figure S1 in Supplementary Material. The 3D structures of the inhibitors (1-methylamine-1-benzyl-cyclopentane, (S)-2-[(R)-3-amino-4-(2-fluoro-phenyl)-butyryl]-1,2,3,4-tetrahydro-isoquinoline-3-carboxylic acid amide, 7-benzyl-1,3-dimethyl-8-piperazin-1-yl-3,7-dihydro-purine-2,6-dione and 8-[(3~{R})-3-azanylpiperidin-1-yl]-7-[(2-bromophenyl)methyl]-1,3-dimethyl-purine-2,6-dione) were retrieved from the PDB chemical component repository and underwent the docking simulation study and the rescoring procedures. As shown in Figure S2 in Supplementary Material, all the computed poses were in strong agreement with the crystallographic data supporting the case-specific reliability of the procedure in computing the binding architectures of the ligands.

**Figure 5:**
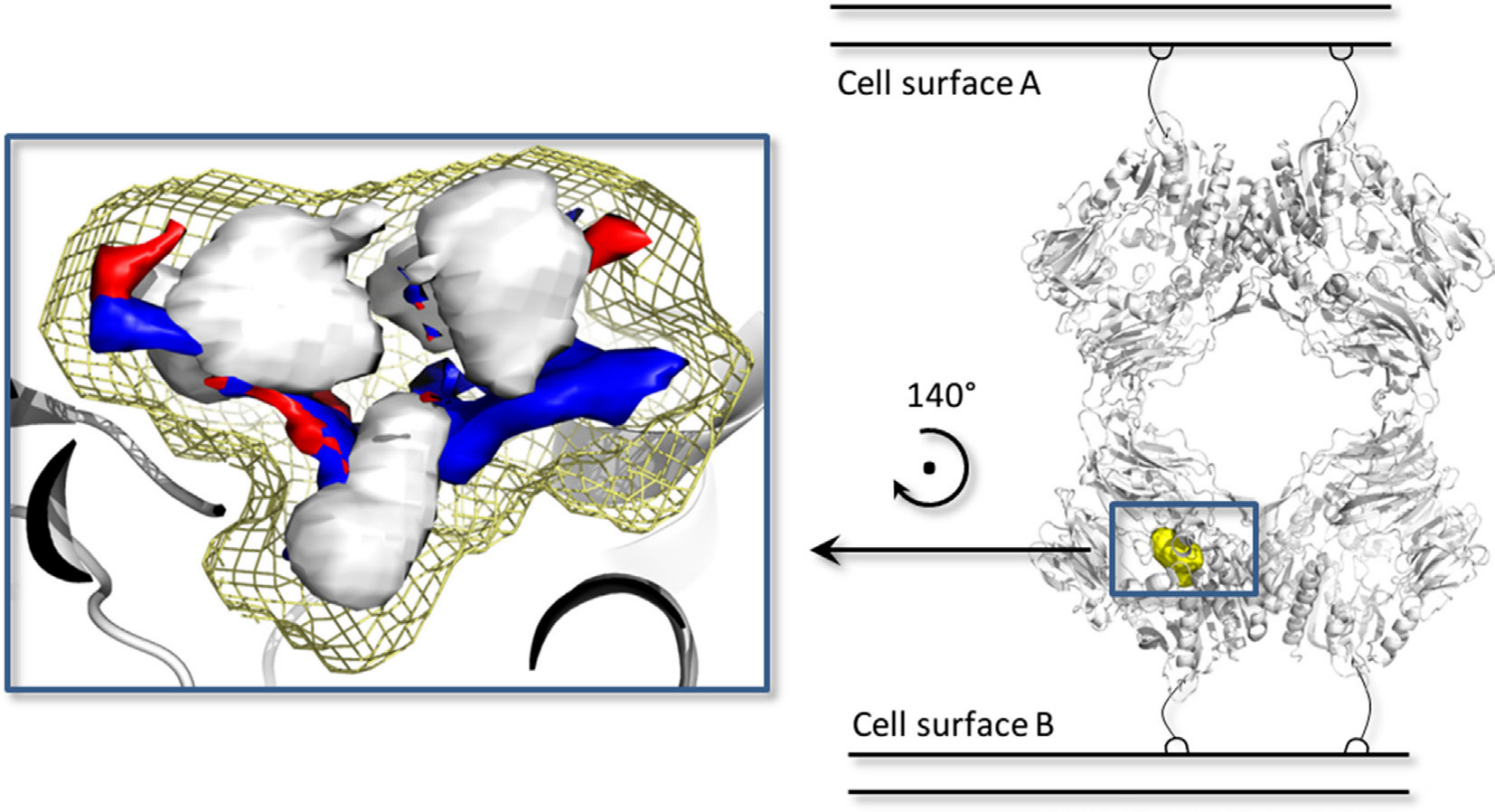
The biological assembly of the porcine dipeptidyl peptidase IV (DPP-IV) (PDB ID 1ORW) (26) is represented in cartoon on the right side, while a close up of pharmacophoric environment of the binding site is represented on the left. The yellow mesh retraces the shape of the binding site, while the white, blue, and red contours identify regions sterically and energetically favorable for hydrophobic, hydrogen bond donor and hydrogen bond acceptor groups, respectively.

**Figure 6:**
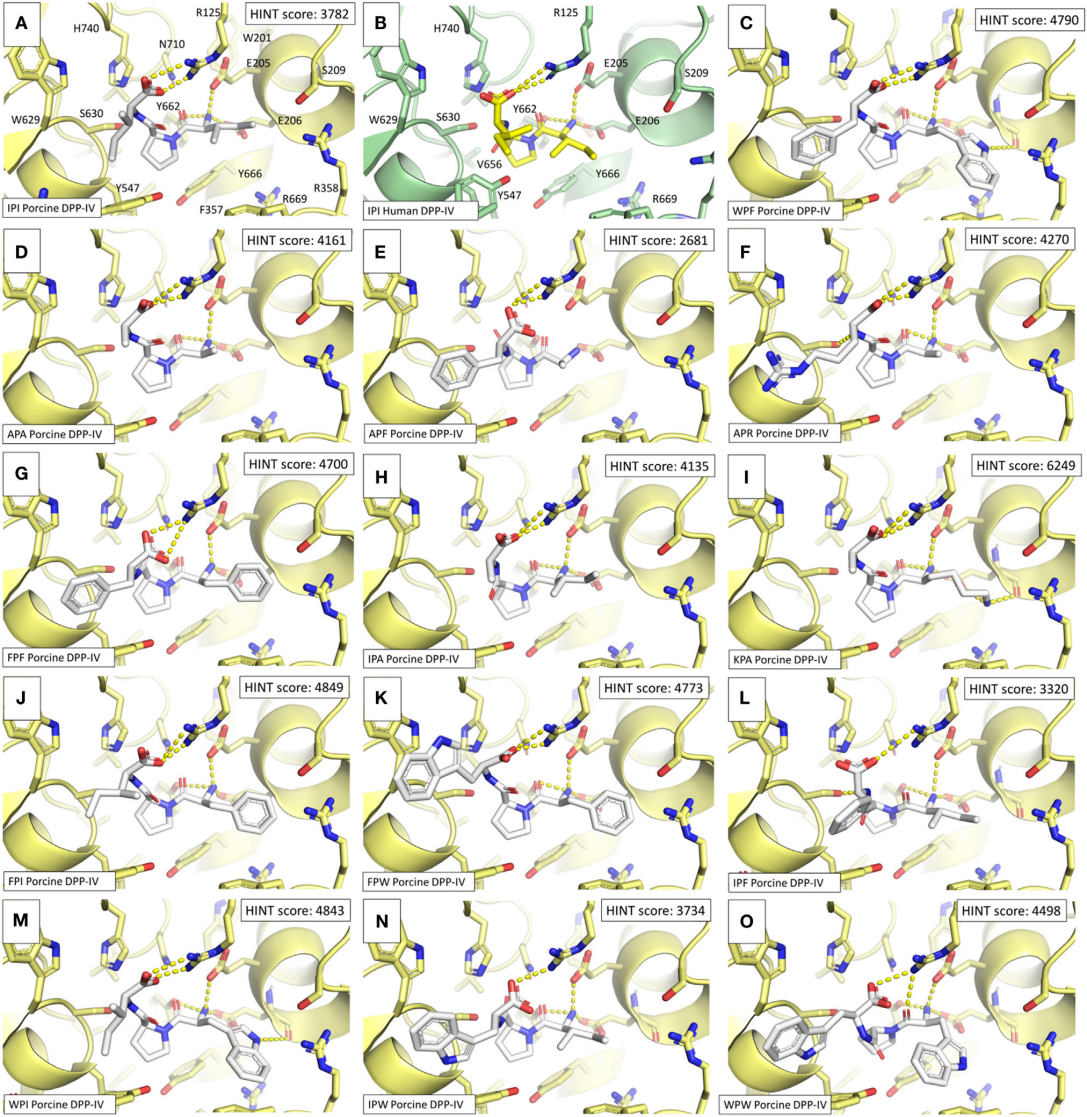
Binding architectures of dipeptidyl peptidase IV (DPP-IV) inhibitory peptides. The protein is represented in cartoon while peptides and amino acid side chains are represented in sticks. Yellow dotted lines indicate the protein–peptide polar interactions. **(A)** Ile-Pro-Ile. **(B)** Crystallographic pose of Ile-Pro-Ile within human DPP-IV [PDB structure 1WCY (37)]. **(C)** Trp-Pro-Phe. **(D)** Ala-Pro-Ala. **(E)** Ale-Pro-Phe. **(F)** Ala-Pro-Arg. **(G)** Phe-Pro-Phe. **(H)** Ile-Pro-Ala. **(I)** Lys-Pro-Ala. **(J)** Phe-Pro-Ile. **(K)** Phe-Pro-Trp. **(L)** Ile-Pro-Phe. **(M)** Trp-Pro-Ile. **(N)** Ile-Pro-Trp. **(O)** Trp-Pro-Trp.

**Figure 7:**
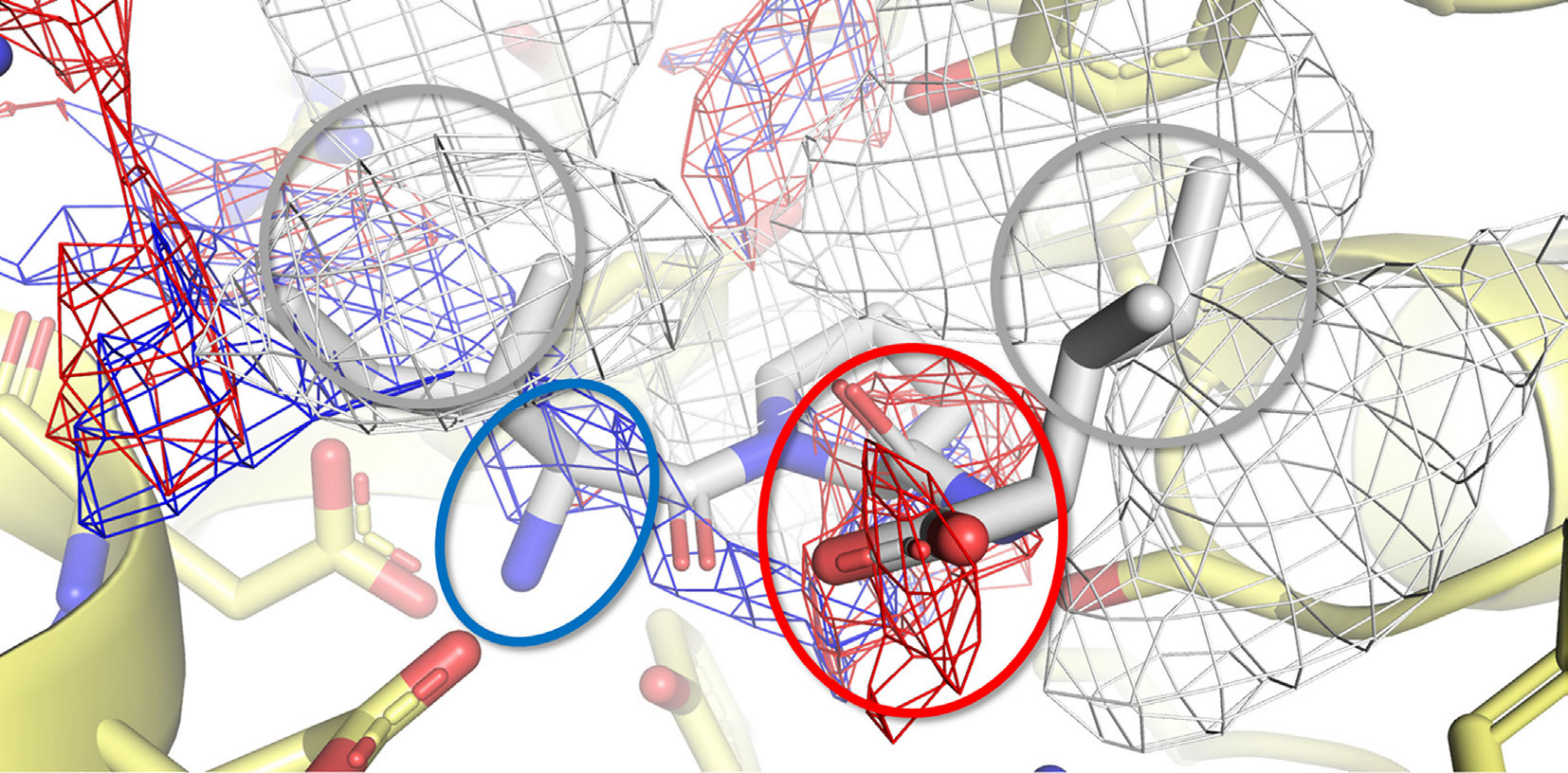
Calculated binding pose of Ile-Pro-Ile within the active site of porcine dipeptidyl peptidase IV (DPP-IV). The protein is represented in cartoon, while the amino acids constitutive of the binding site (yellow) and Ile-Pro-Ile (white) are shown in sticks. The white, blue, and red meshes identify regions sterically and energetically favorable for hydrophobic, hydrogen bond donor and hydrogen bond acceptor groups, respectively. The proper placement of the Ile-Pro-Ile groups in respect to the pharmacophoric requirements of the pocket is highlighted with rings. Specifically, gray indicates hydrophobic regions, while red and blue indicate regions suitable to receive hydrogen bond acceptor and donor groups, respectively.

In addition, a pharmacophoric analysis of the porcine DPP-IV-binding site was carried out to map the space available for ligands in terms of hydrophobic and hydrophilic environment distributions. As shown in Figure 5, the ligand-binding sites showed an environment mainly able to receive hydrophobic and H-bond donor groups, with a limited space suitable for receiving H-bond acceptor groups. The binding architecture of the well-known DPP-IV inhibitory peptide, Ile-Pro-Ile, was calculated along with that of the other DPP-IV inhibitory peptide analogs. As shown in Figures 6A,B, the computed architecture of Ile-Pro-Ile was found to be in strong agreement with the crystallographic pose observed within human DPP-IV (37). In particular, both the ligand arrangement and the polar interactions network computed by the procedure were found to be consistent with those reported in the crystallographic data currently available (Figures 6A,B). This result further confirmed the reliability of the procedure. In addition, the binding pose of Ile-Pro-Ile complied with the pharmacophoric requirements of the binding pocket as the two hydrophobic side chains of Ile were found to be arranged into the hydrophobic space available for ligands, while the C- and N-terminal groups were found to be arranged into regions suitable to receive H-bond acceptor and donor groups, respectively (Figure 7). All the other inhibitory peptides were found to adopt an architecture of binding comparable to Ile-Pro-Ile. In addition, with other peptides, polar interactions between Arg125, Glu205, Glu206, and Tyr662 and Ile-Pro-Ile were also observed (Figure 6).

**Figure 8:**
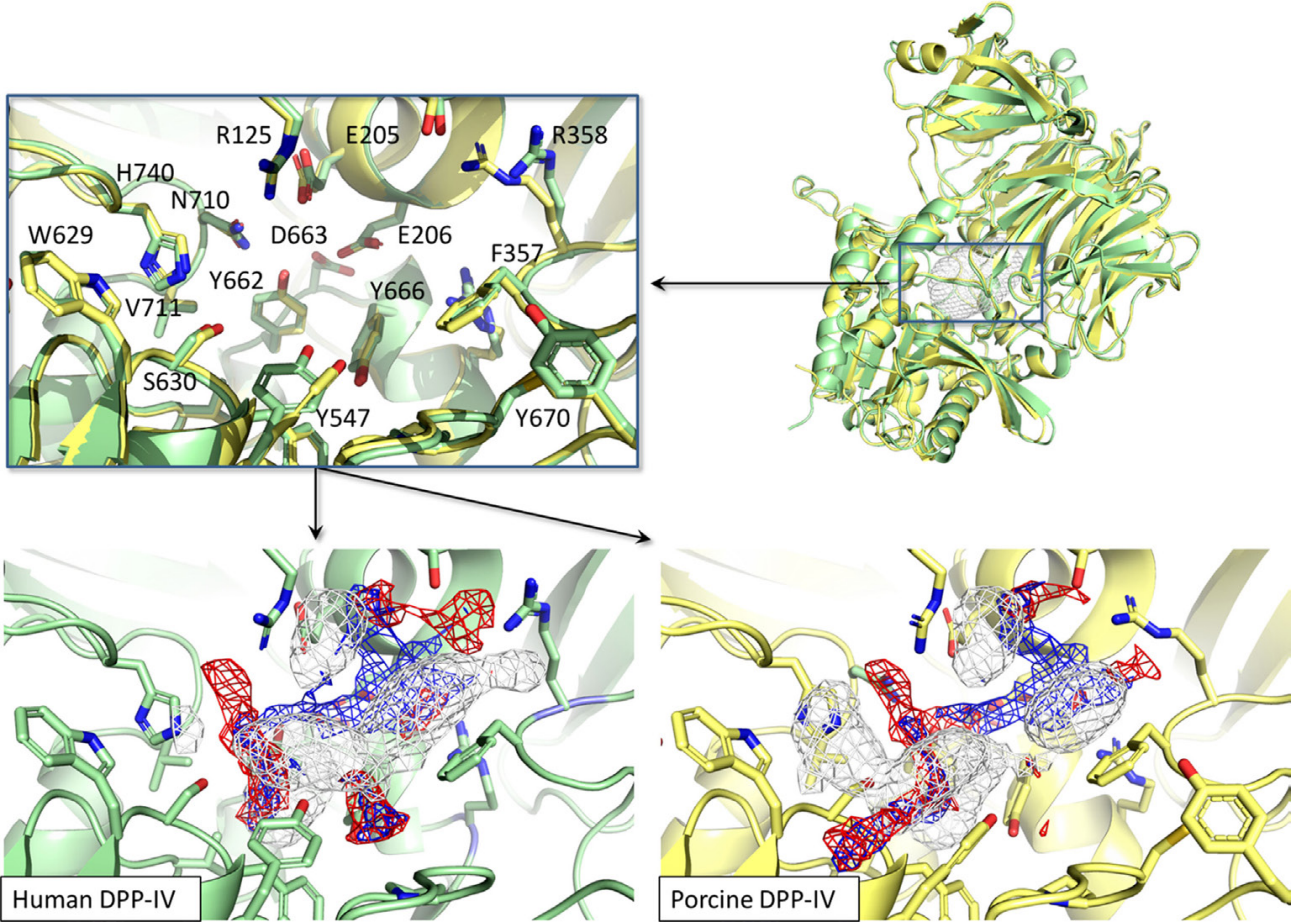
Structural and pharmacophorical comparison between porcine [PDB ID 1ORW (26)] and human [PDB ID 1WCY (37)] dipeptidyl peptidase IV (DPP-IV). Proteins are represented in cartoon while amino acids constitutive of the binding site are represented in sticks. The white, blue, and red meshes identify regions sterically and energetically favorable for hydrophobic, hydrogen bond donor and hydrogen bond acceptor groups, respectively.

The binding site of porcine DPP-IV was compared to that of the human orthologous. The porcine and human enzymes have an identical length of 766 residues and the overall sequence identity is 88% (26) with a strongly conserved 3D organization (Figure 8). In relation to the catalytic site, those residues forming the space available for ligands showed 100% conservation in terms of residue composition and organization (Figure 8). Consequently, the pharmacophoric fingerprints of the two binding pockets were found to be highly comparable (Figure 8).

## Discussion

The ability of Pro-containing tripeptides to inhibit DPP-IV *in vitro* was investigated. It has been suggested that Pro-containing peptides are relatively stable to gastrointestinal enzymes (38). Specific Pro-containing tripeptides may also be bioavailable in humans. For instance, Ile-Pro-Pro and Val-Pro-Pro have been identified in human sera following the ingestion of a milk beverage enriched in lactotripeptides (39). Ile-Pro-Ile is the most potent DPP-IV inhibitory peptide identified to date in the literature (5). Therefore, a MAPS-based approach was employed herein to design peptide analogs of Ile-Pro-Ile with the view to (i) study the role of tripeptide structures on DPP-IV inhibition and (ii) identify novel potent inhibitors of DPP-IV. A range of *in silico* methodologies including DOE, QSAR modeling, and molecular docking were used to design and study peptide analogs of Ile-Pro-Ile.

Several structural and physicochemical parameters of molecules have been shown to affect their ability to inhibit DPP-IV (40). The hydrophobicity of amino acids located within specific positions of peptides is thought to be of importance for the inhibition of DPP-IV (9, 41, 42). The QSAR model (Eq. 1) demonstrated that the hydrophobicity (*v*_3_) of the amino acids at the N-terminal and next to the C-terminal positions of peptides played a role in their DPP-IV inhibitory properties (Figure 2A). For tripeptides, the hydrophobicity (*v*_3_) of the second amino acid was shown to be linked with DPP-IV inhibition by the peptides (Eq. 2; Figure 2B). The models (Eqs 1 and 2) developed here were all significant (*p* < 0.05). However, they had limitations in terms of their predictive ability for DPP-IV inhibitory properties of peptides (Table 2; Figure 2C). Similar results have been obtained during the development of a QSAR model, which showed differences between predicted and experimental DPP-IV IC_50_ values of peptides (17). Similarly, with other metabolic enzymes such as ACE, major differences between QSAR predicted and experimental IC_50_ values have been reported for peptides on a number of occasions (43, 44). These differences are likely due to the fact that physicochemical parameters, other than those that are used to build the models, may play an important role in the inhibitory properties studied.

Other than hydrophobicity, aromaticity and steric hindrance of amino acid residues may play a role in the ability of peptides to inhibit DPP-IV. In a recent review paper on DPP-IV inhibitory properties of molecules, Ojeda-Montes et al. (40) suggested that potent DPP-IV inhibitors included molecules possessing aromatic rings, which can establish hydrophobic interactions with the S_1_ subsite of the DPP-IV active site. Earlier studies have reported on the existence of exclusion volumes in the S_1_ pocket of DPP-IV (45). These exclusion volumes may be responsible for the limited access of bulky amino acids residues to the active site of DPP-IV. This may prevent interactions between certain peptides and the active site of DPP-IV and/or the entry path to the catalytic site. Nevertheless, based on the molecular docking outcomes, all tripeptides studied herein were predicted to interact in a very similar manner with the active site of DPP-IV (Figure 6), and no evident steric interferences due to the aromatic or aliphatic residues in positions Xaa_1_ and Xaa_3_ were found. This finding is consistent with the availability of space to arrange side chains at positions #1 and #3 as both subsites are exposed to the bulk solvent with a large capability to arrange sidechains reducing steric interferences. On the other side, the Xaa_2_ position is likely to be more affected by steric restraints being buried into the protein core with a limited capability to accept bulky side chains (Figure S3 in Supplementary Material). On this basis, the discrepancies between the calculated and experimental inhibitory activity of the peptides considered herein might be due to additional factors beyond the inherent capability to interact with the binding site. This may relate to the capability to go through the entry path of the binding site and/or the capability of peptide binding to induce molecular rearrangement of DPP-IV, which in turn may affect enzymatic activity.

The % DPP-IV inhibition at 500 µM measured with tripeptides from set #1 confirmed that the presence of a Pro at position 2 was important to observe substantial inhibition of DPP-IV (Figures 2A,C). In fact, all tripeptides from sets #1 and 2, which possessed a Pro in position 2 inhibited DPP-IV while peptides possessing a different amino acid in position 2 were not able to inhibit DPP-IV or were not potent DPP-IV inhibitors (Figures 2A,C; Table 2). These results are consistent with earlier findings reporting on the features of potent (IC_50_ < 100 µM) DPP-IV inhibitory tripeptides, which also revealed the occurrence of a Pro residue at position 2 (5). Other analogs of Ile-Pro-Ile have been shown earlier to be relatively potent inhibitors of DPP-IV (Table S1 in Supplementary Material). For instance, Ile-Pro-Ala, also known as β-lactosin A (an antihypertensive peptide), had previously been reported for its ability to inhibit DPP-IV (46). The DPP-IV IC_50_ value of Ile-Pro-Ala reported herein was slightly lower than that reported by Tulipano et al. (46), i.e., 28.3 (Table 2) vs. 49.0 µM. However, this was consistent with the DPP-IV IC_50_ value of Ile-Pro-Ile, which was 3.9 vs. 7 µM (46). Several novel peptides displaying a DPP-IV IC_50_ value < 100 µM (Ala-Pro-Ala, Ala-Pro-Phe, Lys-Pro-Ala, Phe-Pro-Ile, Phe-Pro-Trp, and Ile-Pro-Trp, Table 2) were reported herein for the first time. The presence of a Pro residue at position 2 has often been associated with DPP-IV inhibition. However, it is not a guarantee for high DPP-IV inhibitory potency (Table 2; Table S1 in Supplementary Material). The amino acids located at the N- and C-terminal side of tripeptides also appear to play an important role in their DPP-IV inhibitory properties. This can be seen with the variation in the DPP-IV IC_50_ value of peptides differing in either their N- or C-terminal amino acid (Table 2).

Ile-Pro-Ile possesses the structure of preferred DPP-IV inhibitory substrates. Incubation of Ile-Pro-Ile with DPP-IV was reported to induce its breakdown over time. For instance, after 36 min incubation, 37% of the initial amount of Ile-Pro-Ile was cleaved by DPP-IV (47). These results are in contrast with our findings, showing no hydrolysis of Ile-Pro-Ile following 30 min incubation with DPP-IV, which is consistent with earlier reports (21, 24). Other tripeptides (Ala-Pro-Ala, Ala-Pro-Phe, Ala-Pro-Arg, Ile-Pro-Ala, Lys-Pro-Ala, Phe-Pro-Ile, and Trp-Pro-Ile) studied herein, which possess the structure of preferred DPP-IV inhibitory substrates, were also not hydrolyzed by DPP-IV after 30 min incubation (Table 2; Figure 4). In contrast, Phe-Pro-Phe, Phe-Pro-Trp, Ile-Pro-Phe, Ile-Pro-Trp, Trp-Pro-Phe, and Trp-Pro-Trp were partly hydrolyzed by DPP-IV during 30 min incubation. Substrate-type inhibitors are generally susceptible to hydrolysis by the enzyme (48). Several milk protein-derived peptides with a penultimate Pro have been shown to be degraded during incubation with DPP-IV (21, 49, 50). An earlier study has demonstrated that the extent of cleavage of peptides possessing the structure of DPP-IV substrates depended on the enzyme to substrate ratio (E:S) and the peptide sequence (21). A higher E:S generally resulted in higher extents of peptide cleavage. This may explain the differences seen in the extent of cleavage of Ile-Pro-Ile between the study from Rahfeld et al. (47) and our results. Surprisingly, Ile-Ala-Ile, which also possesses the structure of preferred DPP-IV substrates, could not significantly inhibit DPP-IV (<10% DPP-IV inhibition at 500 µM). Further investigation is warranted to explain this result.

As expected, most peptides with a Pro at position 2 were able to bind the active site of DPP-IV as demonstrated by their competitive mode of inhibition (Table 2). However, peptides possessing a Trp at their N-terminal position, i.e., Trp-Pro-Phe, Trp-Pro-Ile, and Trp-Pro-Trp were mixed-type inhibitors of DPP-IV. Several peptides having a Trp residue at their N-terminal position have been shown to be non-competitive inhibitors of DPP-IV (18, 22, 51). It has been suggested in earlier studies that alternative binding sites, differing from the active site of DPP-IV exist. The outcomes of molecular docking studies suggested that a number of peptides with a Trp N-terminus can bind secondary sites of DPP-IV. The location of this secondary binding site is nearby the active site of DPP-IV (51, 52). Pro-Phe, Trp-Pro-Ile, and Trp-Pro-Trp possess the structure of preferred substrates of DPP-IV (i.e., Pro residue at position 2). This is further confirmed by the fact that Trp-Pro-Phe and Trp-Pro-Trp were hydrolyzed by DPP-IV (Table 2). The mixed mode of inhibition of the peptides studied herein may arise from the fact that Trp-Pro-Phe, Trp-Pro-Ile, and Trp-Pro-Trp are also able to bind both to the active and secondary sites of DPP-IV.

Molecular docking showed that Ile-Pro-Ile and its associated peptide analogs established similar interactions with the active site of porcine DPP-IV (Figure 6). These interactions are consistent with those (i.e., with Glu205, Glu206, Tyr662) that have been demonstrated earlier with the S_1_ pocket of the active site of DPP-IV (40). Nevertheless, there was no correlation between the computed scores and the experimentally determined DPP-IV IC_50_ values (Figure 6; Table 2). Bearing in mind that the HINT score correlates with the free energy of binding (29), this result might suggest that the energy of interaction within the catalytic site of DPP-IV may have a secondary role in determining inhibition of the enzyme. This may particularly be the case here since the peptides also possess the structure of preferred DPP-IV substrates and, therefore, the mechanisms of inhibition may be more complex than a simple binding of peptides to the active site. This hypothesis is further supported by the unexpected stability of the peptides during incubation with DPP-IV (Table 2; Figure 4). It appears that the manner in which peptides bind to the active site of DPP-IV may protect them from extensive degradation. It is interesting to note that all peptides cleaved by DPP-IV possess bulky amino acid residues (i.e., Trp and Phe) at their C-terminal side.

The results herein are relevant to glycemic regulation and potentially food intake in humans. This is particularly the case as the study herein reports on potent DPP-IV inhibitory peptides (Ile-Pro-Ile and Ile-Pro-Ala) having the characteristics of peptides, which have previously been shown to be relatively stable to gastrointestinal enzymes (53, 54). Several of these peptides were also shown to be relatively stable to DPP-IV (Table 2; Figure 4). Therefore, they may be resistant to the action of brush border DPP-IV in the intestine and protect incretins from early degradation by DPP-IV. There was 100% conservation for the composition and organization of residues forming the space available for ligands within the crystal structures of porcine and human DPP-IV (Figure 8). Therefore, it may be assumed that peptides able to interact with the porcine DPP-IV active site may also interact with that of the human ortholog. As a consequence, inhibition of human DPP-IV activity may be expected for the most potent peptides studied herein. Nonetheless, due to the non-conserved residues at other regions in DPP-IV, effects in reaching the binding site and/or in regulating the protein dynamics, and the reaction kinetics cannot be excluded. Accordingly, differences in the magnitude of the inhibition between the two orthologous may occur. In this regard, it is worth mentioning, however, that short peptides (<5 residues) have been shown to inhibit human and porcine DPP-IV in a similar manner (55). The ability of peptide analogs of Ile-Pro-Ile to play a role as DPP-IV inhibitors in humans needs to be evaluated during human intervention studies.

In recent studies, the utilization of Pro-rich protein substrates has been employed as a strategy to develop relatively potent DPP-IV inhibitory hydrolyzates. The 1 kDa permeate of an ultrafiltration fraction from a porcine skin gelatin hydrolyzate had a DPP-IV IC_50_ value of 1.50 mg mL^−1^ (56). Salmon gelatin hydrolyzates having DPP-IV IC_50_ values between 0.08 ± 0.01 and 0.71 ± 0.09 mg mL^−1^ have been reported (57). Wheat gluten hydrolyzates, which are also rich in Pro-containing peptides, had DPP-IV IC_50_ values of 0.136 (58) and 0.24 ± 0.02 to 0.66 ± 0.06 mg mL^−1^ (59). Food (milk, porcine skin gelatin, and wheat gluten) hydrolyzates were shown to contain numerous tripeptides with a Pro at position 2, some of which had DPP-IV IC_50_ values < 100 µM (53, 54, 56, 58–61). While the effects of these hydrolyzates in humans have not been evaluated, they may represent natural alternatives to synthetic drugs for glycemic regulation.

## Conclusion

Within this work, the utilization of MAPS has been described for the study of analogs of Ile-Pro-Ile, the most potent DPP-IV inhibitory peptide identified to date. While relatively potent DPP-IV inhibitory peptides were identified, none were as potent as Ile-Pro-Ile. This study has confirmed the importance of a Pro residue in position 2 of tripeptides in displaying potent DPP-IV inhibitory properties. Molecular docking showed very similar interactions between Ile-Pro-Ile and its associated peptide analogs. However, the HINT scores did not correlate with the DPP-IV IC_50_ values obtained experimentally. This result, together with the surprisingly high stability of Ile-Pro-Ile and its associated peptide analogs to the hydrolytic action of DPP-IV suggests that complex mechanisms are involved in the inhibition of DPP-IV by these peptides. The peptides investigated in this study may have potential applications for glycemic management and energy homeostasis in humans.

## Author Contributions

AN, LD, SP, RF, PC, and GG contributed to the conception and design of the study. AN, LD, and SP carried out the experimental work, data analysis, and interpretation. AN and LD wrote the first draft of the manuscript. SP, PC, GG, and RF critically revised the manuscript. All authors listed have directly contributed to the manuscript, revised, read, and approved the submitted version.

## Conflict of Interest Statement

The authors declare that the research was conducted in the absence of any commercial or financial relationships that could be construed as a potential conflict of interest.

## Footnotes

^1^http://www.rcsb.org (Accessed: January 27, 2018).

^2^www.certara.com (Accessed: February 26, 2017).

^3^http://www.moldiscovery.com (Accessed: January 8, 2017).

^4^http://www.pymol.org (Accessed: March 6, 2017).
