# Supplementary material for: In Silico Approaches Applied to the Study of Peptide Analogs of Ile-Pro-Ile in Relation to Their Dipeptidyl Peptidase IV Inhibitory Properties
Source: Front Endocrinol (Lausanne). 2018 Jun 14;9:329. doi: 10.3389/fendo.2018.00329 (PMC6010526; doi:10.3389/fendo.2018.00329)
Supplement: Supplementary file 1 [file presentation_1.PDF]

Supplementary data

Supplementary Table S1. Dipeptidyl peptidase IV (DPP-IV) half maximal inhibitory concentration (IC_{50}) of tripeptides reported in the literature.

Peptide sequence[1]	DPP-IV IC_{50} (µM)	Mode of inhibition	Reference
IPI	3.2; 3.5	competitive	(1,2)
VPL	15.8	competitive	(2,3)
GPX	43.5	na	(4)
IPA	49.0	competitive	(5)
LPQ	56.7	competitive	(6)
IPM	69.5	competitive	(7)
QPG	70.9	competitive	(6)
QPF	71.7	competitive	(6)
SPQ	78.9	competitive	(6)
QPQ	79.8	competitive	(6)
LPQ	56.7; 82.0	na	(6,8)
LPL	241.4	competitive	(1)
YPY	243.7	competitive	(1)
PPL	390.1	na	(9)
GPM	417.9	na	(10)
LPP	563.3	non-competitive	(7)
GPV	794.8	na	(10)
PPG	2252.7	na	(9)
GPA	5030.0	na	(11)

[1]Peptide sequences abbreviated with the one letter amino acid code.

na: not available

Comparison between computational geometries of binding and crystallographic poses of dipeptidyl peptidase IV (DPP-IV) inhibitors

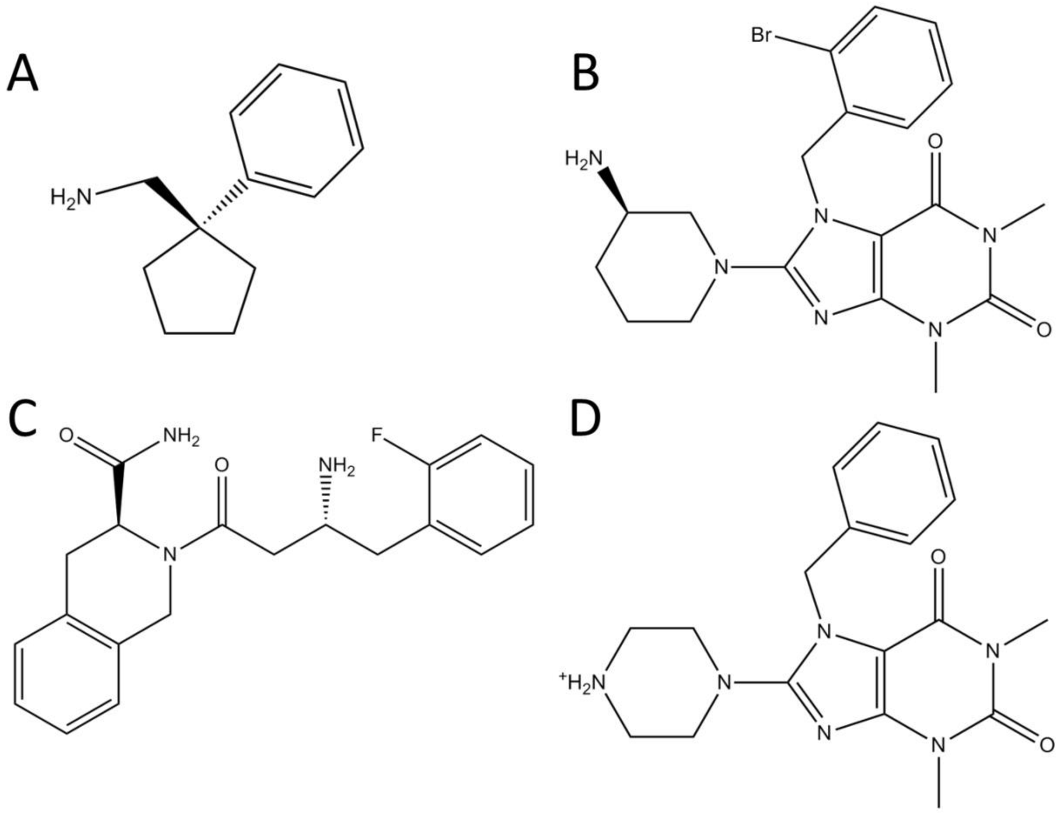

Figure S1. The chemical structures of compounds taken as reference are shown. **A.** 1-methylamine-1-benzyl-cyclopentane. **B.** 8-[(3~{R})-3-azanylpiperidin-1-yl]-7-[(2-bromophenyl)methyl]-1,3-dimethyl-purine-2,6-dione **C.** (S)-2-[(R)-3-amino-4-(2-fluoro-phenyl)-butyryl]-1,2,3,4-tetrahydro-isoquinoline-3-carboxylic acid amide. **D.** 7-benzyl-1,3-dimethyl-8-piperazin-1-yl-3,7-dihydro-purine-2,6-dione.

The compounds taken as reference for the procedure validation are shown in Figure S1. To check the procedure reliability in predicting the geometries of binding, the computed poses of benchmark compounds reported in Figure S1 have been compared to those obtained in the respective crystallographic studies. As shown in Figure S2 all the calculated geometries of binding have been found consistent with the crystallographic poses, confirming the reliability of the procedure.

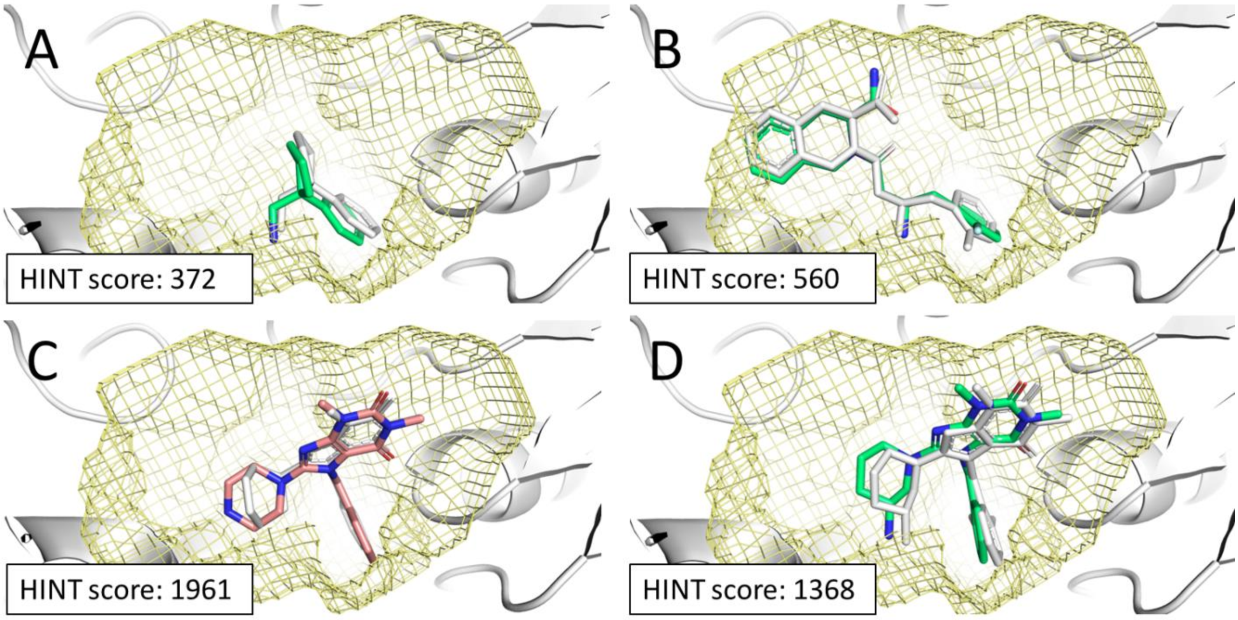

Figure S2. Comparison between the computational geometries of binding and the crystallographic poses (in white color) of the reference compounds. **A.** 1-methylamine-1-benzyl-cyclopentane (PDB ID 007) from the PDB structure 2BUA (12). **B.** (S)-2-[(R)-3-amino-4-(2-fluoro-phenyl)-butyryl]-1,2,3,4-tetrahydro-isoquinoline-3-carboxylic acid amide (PDB ID 008) from the PDB structure 2BUC (12). **C.** 7-benzyl-1,3-dimethyl-8-piperazin-1-yl-3,7-dihydro-purine-2,6-dione (PDB ID SC3) from the PDB structure 2AJ8 (13) and **D.** 8-[(3~{R})-3-azanylpiperidin-1-yl]-7-[(2-bromophenyl)methyl]-1,3-dimethyl-purine-2,6-dione (PDB ID 6Z8) from the PDB structure 5LLS (14).

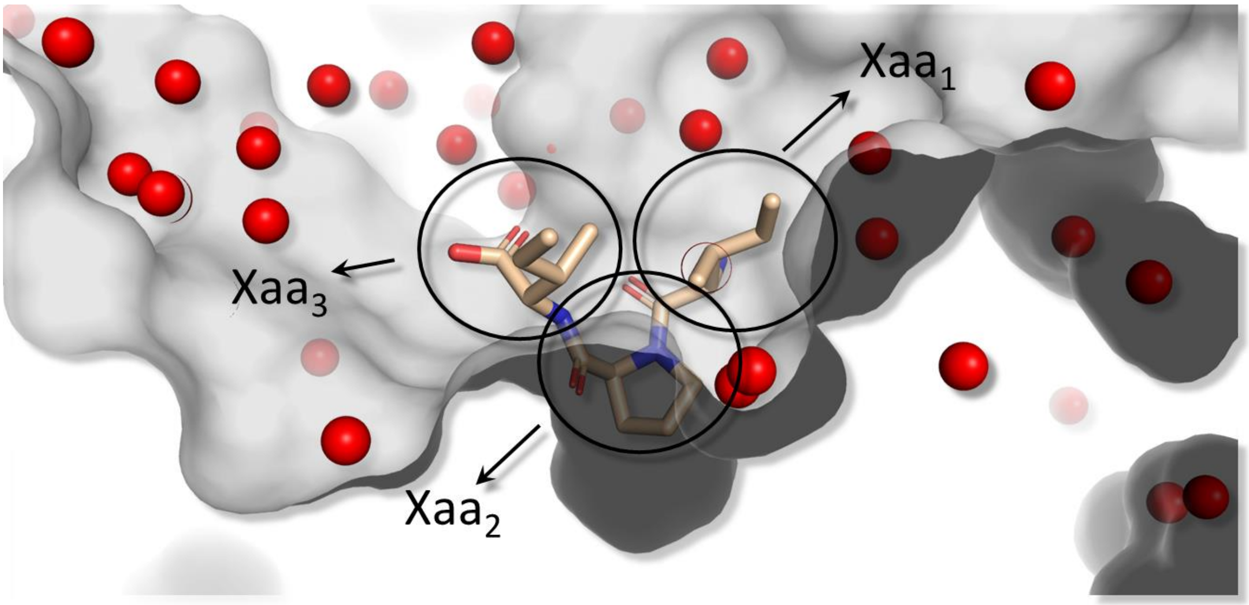

Figure S3. Representation of crystallographic pose of Ile-Pro-Ile (PDB code 1WCY) (15). The white surface retraces the shape of the dipeptidyl peptidase IV (DPP-IV) binding site, red spheres indicate water molecules forming the bulk solvent and Ile-Pro-Ile is represented in yellow sticks. Xaa is an amino acid residue.

References

1. Nongonierma AB, and FitzGerald RJ, Susceptibility of milk protein-derived peptides to dipeptidyl peptidase IV (DPP-IV) hydrolysis. *Food Chem* (2014) 145(15): 845-852. doi: 10.1016/j.foodchem.2013.08.097
2. Rahfeld J, Schierborn M, Hartrodt B, Neubert K, and Heins J, Are diprotin A (Ile-Pro-Ile) and diprotin B (Val-Pro-Leu) inhibitors or substrates of dipeptidyl peptidase IV? *Biochim Biophys Acta, Prot Struct Mol Enzymol* (1991) 1076(2): 314-316. doi: 10.1016/0167-4838(91)90284-7
3. Umezawa H, Aoyagi T, Ogawa K, Naganawa H, Hamada M, and Takeuchi T, Diprotins A and B, inhibitors of dipeptidyl aminopeptidase IV, produced by bacteria. *J Antibiot* (1984) 37(4): 422-425. doi: 10.7164/antibiotics.37.422
4. Hsu K-C, Tung Y-S, Huang S-L, and Jao C-L, Dipeptidyl peptidase-IV inhibitory activity of peptides in porcine skin gelatin hydrolysates. in: B. Hernández-Ledesma, (Ed.), Bioactive Food Peptides in Health and Disease, In Tech, http://www.intechopen.com/books/bioactive-food-peptides-in-health-and-disease/dipeptidyl-peptidase-iv-inhibitory-activity-of-peptides-in-porcine-skin-gelatin-hydrolysates. Doi: 10.5772/51264, 2013, pp. 205-218.
5. Tulipano G, Sibilia V, Caroli AM, and Cocchi D, Whey proteins as source of dipeptidyl dipeptidase IV (dipeptidyl peptidase-4) inhibitors. *Peptides* (2011) 32(4): 835-838. doi: 10.1016/j.peptides.2011.01.002
6. Taga Y, Hayashida O, Kusubata M, Ogawa-Goto K, and Hattori S, Production of a novel wheat gluten hydrolysate containing dipeptidyl peptidase-IV inhibitory tripeptides using ginger protease. *Biosci Biotech Biochem* (2017) 81(9): 1823-1828. doi: 10.1080/09168451.2017.1345615
7. Nongonierma AB, and FitzGerald RJ, Structure activity relationship modelling of milk protein-derived peptides with dipeptidyl peptidase IV (DPP-IV) inhibitory activity. *Peptides* (2016) 791-7. doi: 10.1016/j.peptides.2016.03.005
8. Uenishi H, Kabuki T, Seto Y, Serizawa A, and Nakajima H, Isolation and identification of casein-derived dipeptidyl-peptidase 4 (DPP-4)-inhibitory peptide LPQNIPPL from gouda-type cheese and its effect on plasma glucose in rats. *Int Dairy J* (2012) 22(1): 24-30. doi: 10.1016/j.idairyj.2011.08.002
9. Lafarga T, O'Connor P, and Hayes M, Identification of novel dipeptidyl peptidase-IV and angiotensin-I-converting enzyme inhibitory peptides from meat proteins using *in silico* analysis. *Peptides* (2014) 5953-62. doi: 10.1016/j.peptides.2014.07.005
10. Jin Y, Yan J, Yu Y, and Qi Y, Screening and identification of DPP-IV inhibitory peptides from deer skin hydrolysates by an integrated approach of LC–MS/MS and *in silico* analysis. *J Funct Foods* (2015) 18344-357. doi: 10.1016/j.jff.2015.07.015
11. Hatanaka T, Kawakami K, and Uraji M, Inhibitory effect of collagen-derived tripeptides on dipeptidylpeptidase-IV activity. *J Enzyme Inhib Med Chem* (2014) 29(6): 823-828. doi: 10.3109/14756366.2013.858143
12. Nordhoff S, Cerezo-Gálvez S, Feurer A, Hill O, Matassa VG, Metz G, Rummey C, Thiemann M, and Edwards PJ, The reversed binding of β-phenethylamine inhibitors of DPP-IV: X-ray structures and properties of novel fragment and elaborated inhibitors. *Bioorg Med Chem Lett* (2006) 16(6): 1744-1748. doi: 10.1016/j.bmcl.2005.11.103
13. Engel M, Hoffmann T, Manhart S, Heiser U, Chambre S, Huber R, Demuth H-U, and Bode W, Rigidity and flexibility of dipeptidyl peptidase IV: Crystal structures of and docking experiments with DPP IV. *J Mol Biol* (2006) 355(4): 768-783. doi: 10.1016/j.jmb.2005.11.014
14. Schnapp G, Klein T, Hoevels Y, Bakker RA, and Nar H, Comparative analysis of binding kinetics and thermodynamics of dipeptidyl peptidase-4 inhibitors and their relationship to structure. *J Med Chem* (2016) 59(16): 7466-7477. doi: 10.1021/acs.jmedchem.6b00475
15. Hiramatsu H, Yamamoto A, Kyono K, Higashiyama Y, Fukushima C, Shima H, Sugiyama S, Inaka K, and Shimizu R, The crystal structure of human dipeptidyl peptidase IV (DPPIV) complex with diprotin A. *Biol Chem* (2004) 385(6): 561–564. doi: 10.1515/BC.2004.068
